# Earth's Phosphides in Levant and insights into the source of Archean prebiotic phosphorus

**DOI:** 10.1038/srep08355

**Published:** 2015-02-10

**Authors:** Sergey N. Britvin, Michail N. Murashko, Yevgeny Vapnik, Yury S. Polekhovsky, Sergey V. Krivovichev

**Affiliations:** 1 Institute of Geosciences, St. Petersburg State University, Universitetskaya Nab. 7/9, 199034 St. Petersburg, Russia; 2 Department of Geological and Environmental Sciences, Ben-Gurion University of the Negev, P.O.B. 653, Beer-Sheva 84105, Israel; 3 Nanomaterials Research Center, Kola Science Center of RAS, Fersman Str. 18, 184200 Apatity, Russia

## Abstract

Natural phosphides - the minerals containing phosphorus in a redox state lower than zero – are common constituents of meteorites but virtually unknown on the Earth. Herein we present the first rich occurrence of iron-nickel phosphides of terrestrial origin. Phosphide-bearing rocks are exposed in three localities in the surroundings of the Dead Sea, Levant: in the northern Negev Desert, Israel and Transjordan Plateau, south of Amman, Jordan. Seven minerals from the ternary Fe-Ni-P system have been identified with five of them, NiP_2_, Ni_5_P_4_, Ni_2_P, FeP and FeP_2_, previously unknown in nature. The results of the present study could provide a new insight on the terrestrial origin of natural phosphides – the most likely source of reactive prebiotic phosphorus at the times of the early Earth.

## Phosphides as likely sources of prebiotic phosphorus

Phosphorus, one of life essential elements, exists under current geochemical conditions in the pentavalent state as orthophosphate ion (P^5+^O_4_)^3−^. However, at the times of the early Earth the situation might be very different: recently, phosphite species, (HP^3+^O_3_)^2−^, containing reduced trivalent phosphorus, have been identified in marine sediments related to the Archean era^1^. It was suggested that the phosphorus(III) served as a precursor of prebiotic organophosphorus compounds^2^,^3^, which raises an important question on the origin of reduced phosphorus in the Archean era. The plausible hypothesis assumes that the phosphite ions could be generated by the oxidation of phosphides, oxygen-free minerals containing phosphorus in a redox state lower than zero^4^,^5^.

Phosphide minerals are common accessory phases in meteorites and, to a lesser extent, in lunar rocks, interplanetary dust particles and comets^6^,^7^,^8^. Among eight known natural phosphides, five related to the ternary Fe-Ni-P system are particularly significant from the cosmochemical viewpoint: schreibersite - nickelphosphide, (Fe,Ni)_3_P - (Ni,Fe)_3_P (refs. 6,9), barringerite and its high-pressure polymorph allabogdanite, (Fe,Ni)_2_P (refs. 10,11,12), and melliniite, (Fe,Ni)_4_P (ref. 13). Knowledge of the phase relations in the Fe-Ni-P system substantially contribute to the understanding of the origin of planetesimals in the Solar System^6^,^7^,^14^ and to the prediction of phase transitions in planetary interiors^12^,^14^,^15^. Contrary to meteorites, phosphides have never been found in terrestrial rocks, with the exception of schreibersite found as trace inclusions in native iron from the Disko Island, Greenland^16^, the single find of barringerite in garnet peridotite from China^17^ and a few grains of phosphides found in fulgurites – silicate glasses formed by cloud-to-ground lightning events^18^,^19^.

The almost complete absence of phosphides in the Earth's crust resulted in the conventional view on the extraterrestrial origin of these minerals, hence meteoritic bombardment of the Earth's surface is considered as a likely source of phosphides and therefore of the reactive prebiotic phosphorus in the Archean era^1^,^2^,^3^,^4^,^5^.

## Phosphides in Levant

**Figure 1:**
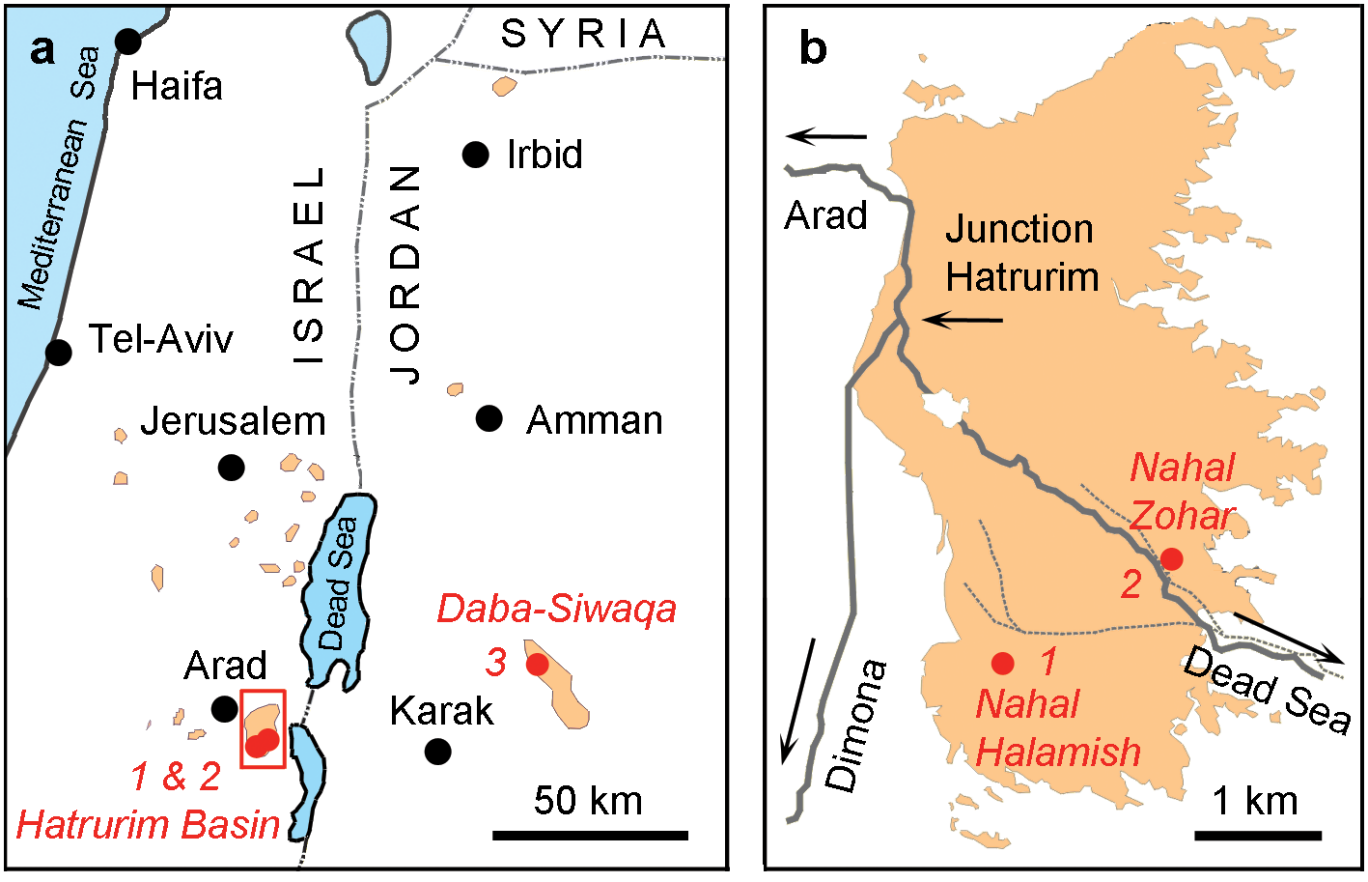
Phosphide occurrences in Hatrurim Formation. a, Location map of Mottled Zone outcrops (indicated as brown areas). Phosphide localities in the Hatrurim basin, Negev Desert (#1 and #2) and Daba-Siwaqa complex, Transjordan Plateau (locality #3) are indicated by red circles. b, Detailed scheme showing positions of phosphide-bearing localities in the Hatrurim basin, Negev Desert. The positions of the localities were determined during the field trips by means of GPS (Michail Murashko and Yevgeny Vapnik). The maps a and b were created in Adobe Photoshop CS5.1.

Phosphide mineralization described herein occurs in the pyrometamorphic rocks related to the Hatrurim Formation, also known as a “Mottled Zone”. This unique geological complex is located in the vicinity of the Dead Sea, Levant, covering large areas in Israel and Jordan (Fig. 1a)^20^,^21^. The Mottled Zone is exposed in a series of outcrops with a total area of ~600 km^2^. The thickness of the formation reaches several hundred meters. The Mottled Zone has attracted scientific interest as a suite of chalky-marly sediments which underwent extensive and repetitive high-temperature (500–1500°C) and low-pressure (down to one bar) metamorphic events^20^,^21^. The sediments were subject to a combustion metamorphism, i.e. pyrometamorphism caused by the spontaneous or stimulated burning of carbonaceous matter. Two popular hypotheses explaining the source of the fired substance have been proposed. The first points out that the primary materials were bituminous sedimentary marls and chalks adjacent to the Mottled Zone^20^,^21^ and the second hypothesis is that the high temperature was a result of a firing of hydrocarbons (preferentially methane) from mud volcano explosions initiated by tectonic activity at the Dead Sea transform fault^22^. Both approaches are based on valuable observations and the exact origin of Hatrurim Formation is still a matter of debates. The age of the major metamorphic event was determined as 2–4 million years meaning that the formation is juvenile from the geological point of view^23^. High temperatures reached during combustion metamorphism resulted in the emergence of paragenetic mineral associations corresponding to the highest metamorphic grades such as pyroxene-hornfels and sanidinite facies, and even in the formation of specific marbles composed of minerals know from cement clinkers, such as larnite, Ca_2_SiO_4_, and hatrurite, Ca_3_SiO_5_ (refs. 20,21). The primary mineralogy of the pyrometamorphic rocks of the Mottled Zone is rather complex itself^20^, but successive post-metamorphic hydrothermal alteration and weathering expanded mineral diversity even further. The total list of minerals known from the Hatrurim Formation approaches 200 species^22^.

**Figure 2:**
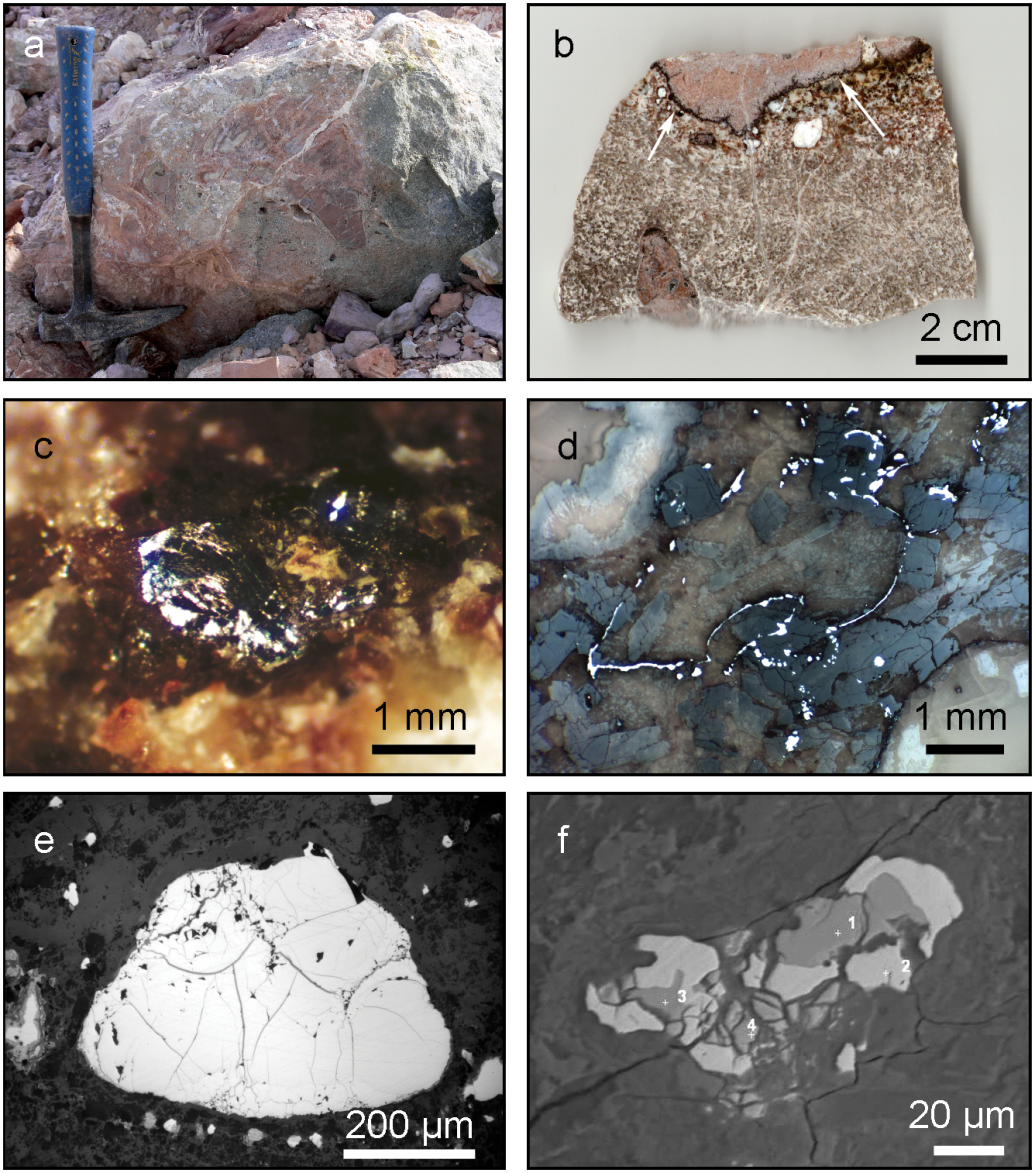
Phosphide associations of the Mottled Zone. a, Brecciated paralava with phosphide mineralization in a quarry in Daba-Siwaqa complex, Transjordan Plateau (locality #3). b, Polished slice of paralava breccia sampled from the quarry in the locality #3. Contact zone between clinopyroxene-anorthite paralava (bottom) and calcined metamorphosed chalk (top) is traced by the chain of black phosphide grains marked by arrows. White patches and veinlets are composed by late hydrothermal aragonite. c, 2.5 mm size grain of phosphides in silicate matrix. Nahal Halamish, Hatrurim basin (locality #1). d, Chains of phosphide grains scattered along the boundaries between silicate grains (locality #3). Polished section, reflected light. e, Grain of barringerite, Fe_2_P, in silicate matrix. Polished section, reflected light. Nahal Zohar, Hatrurim basin (locality #2). f, Zuktamrurite, FeP_2_, (dark areas – points 1,3,4) intergrown with transjordanite, Ni_2_P, (light areas – point 2). Phosphides are embedded into calcite-silicate matrix. Backscattered electron image. Locality #1. Photographs were made by Yevgeny Vapnik (a), Michail Murashko (b,f), Sergey Britvin (c,d,e). Photographs were assembled in Adobe Photoshop CS5.1.

Phosphides were found in the two outcrops within the area of Hatrurim Basin (wadi Nahal Halamish and Nahal Zahar - localities #1 and #2 on Fig. 1b) and in a small quarry exposing subsurface rocks of Daba-Siwaqa complex in Transjordan Plateau (locality #3, Fig. 1a). The first two localities are about 2 km apart, whereas the Transjordan occurrence is ~100 km NE of them, situated on the opposite side of the Dead Sea (Fig. 1a). In spite of large distances between the occurrences, phosphide associations in all three outcrops are similar. The host rocks are represented by “paralavas” (ref. 22) (Fig. 2a) – light-colored remelted carbonate-silicate sediments composed of clinopyroxene, anorthite, wollastonite, melilite and grossular-andradite garnets. Paralavas often contain centimeter- to decimeter-sized brecciated fragments of calcined marls, chalks and phosphorites (Fig. 2b). Late hydrothermal calcite, aragonite and a variety of hydrous calcium alumosilicates fill voids and fractures in paralava breccia. Phosphide grains ranging from a few microns to a few millimeters in size are usually located across the boundaries between brecciated clasts and host paralavas (Fig. 2c–f). Macroscopically, phosphides appear as yellowish-grey to nearly black inclusions in light-colored silicate-carbonate matrix and possess bright metallic luster. The minerals may occur as grains composed of a single-phase phosphide (Fig. 2e) or as complex intergrowths of two to three mineral phases (Fig. 2f). The phosphide grains that are exposed onto the weathered rock surfaces demonstrate different degrees of oxidation being partially or completely replaced by yet unidentified hydrous iron-nickel phosphates. The minerals usually associated with phosphides are pyrrhotite, Fe_1-x_S, troilite FeS, sometimes grains of Ni-free native iron, iron-phosphide eutectic, trevorite (natural spinel NiFe_2_O_4_), hematite, fluorapatite and a natural pyrophosphate CaFe^2+^P_2_O_7_, which is currently under investigation.

**Figure 3:**
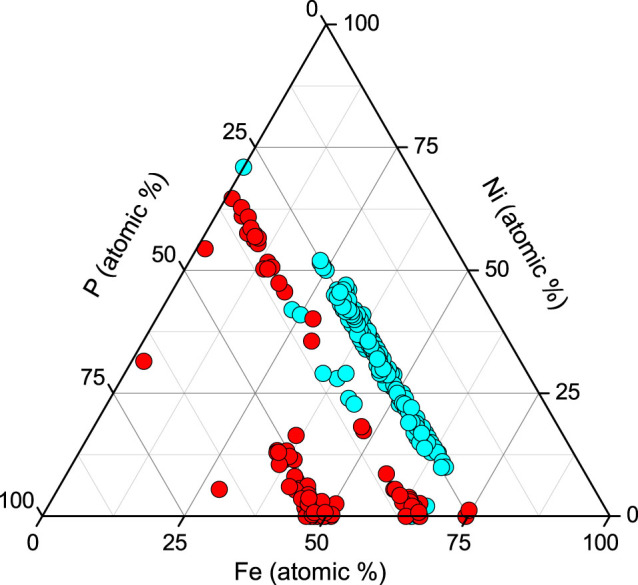
Ternary diagram for the Fe-Ni-P system illustrating compositional ranges of meteoritic phosphides (blue circles) in comparison with terrestrial phosphides from the Hatrurim Formation (red circles). Based on the data from refs 6,7,9,10,13,25,26 and. The diagram was created in Origin 8.6 Academic Edition.

In total, we have identified seven minerals related to the Fe-Ni-P system, of those five are new in nature (Table 1). Representative chemical compositions of the phosphides reported herein are summarized in Tables S1–S7; crystallographic data for negevite, zuktamrurite, murashkoite, halamishite and transjordanite are presented in Tables S8–S12. The general chemical differences between meteoritic and terrestrial (i.e., Hatrurim) phosphides are illustrated by the ternary Fe-Ni-P plot (Fig. 3). It can be seen that minerals from the Hatrurim Formation exhibit much broader range of the metal-to-phosphorus ratios along with the extreme variations in the Fe/Ni ratios. Nickel is known as a mandatory constituent of meteoritic phosphides^6^,^7^,^9^,^10^,^13^,^24^,^25^, whereas the reported finds of terrestrial phosphides are nickel-poor^16^,^17^. It is noteworthy that some of the Hatrurim phosphides, such as halamishite Ni_5_P_4_ and negevite NiP_2_ do contain as low as 2–3% of Fe – the values not reported even in meteoritic phosphides, with the exception of the single grain of Ni_5_P_2_ found in the carbonaceous chondrite Allende^26^. The most likely source of nickel required for the formation of Ni-phosphides was natural oxide spinel trevorite, NiFe_2_O_4_, occurring in the same metamorphic rocks as phosphides. Trevorite, in due course, could be formed during metamorphic transformations of sedimentary marls and chalks which are reported to contain more than 200 ppm of Ni^27^. Note that completely distinct Ni-rich and Ni-free phosphides in the Mottled Zone often coexist in intimate intergrowths (Fig. 2f) that might indicate their rapid in-situ formation under non-equilibrium reducing environment. The temperatures attained in the course of phosphide formation were undoubtedly higher than 1050°C as indicated by the coexistence of phosphide grains with binary schreibersite-iron eutectic (steadite) well known in metallurgy^28^.

The origin of reducing conditions and high temperature needed for the transformation of oxides and phosphates to phosphides could be explained in the general context of the origin of the Mottled Zone^20^,^21^,^22^. It is known that the coal-based reduction of phosphates is widely employed in the industrial production of ferrophosphorus^29^. On the other hand, gaseous methane is known as an effective reductant of phosphates to phosphides^30^. Therefore, combustion of either bituminous rocks^20^,^21^ or methane originated from mud volcanoes^22^ could provide the environment sufficient for the direct reduction of natural phosphates and trevorite into Fe-Ni phosphides. At the same time, local spatial enrichment in nickel needed for the formation of trevorite and phosphides could be achieved via gas transport reactions involving, for instance, highly volatile nickel carbonyl Ni(CO)_4_. The source of phosphorus in phosphides has a straightforward explanation: it could be supplied via the incorporation of phosphorite breccia by the paralava melts. Phosphorite is the major constituent of the sedimentary Mishash Formation (late Campanian age) underlying the rocks of the Mottled Zone, where it forms meter-thick layers interstratified with marls and chalks^21^. Centimeter-sized clasts of metamorphosed phosphorites are common in paralavas, and fine grains of fluorapatite along with CaFeP_2_O_7_ are frequently associated with phosphide grains.

## Earth's *vs*. meteoritic: what was the source of prebiotic phosphorus?

Phosphides, whose meteoritic origin has never been doubted, are arguably considered as likely precursors for prebiotic organophosphorus compounds^1^,^2^,^3^,^4^,^5^. In this respect, the occurrence of geologically juvenile terrestrial phosphides, being interesting itself, could call for the new insight on the source of “phosphide phosphorus” at the era of the early Earth: the Levant findings can provide a reliable model for the formation of the Earth's own prebiotic phosphides rather than meteoritic ones. Following to the present paper, the four conditions required for the formation of terrestrial Fe-Ni phosphides can be formulated: (1) the source of phosphate phosphorus; (2) the source of counterpart transition metals (i.e., Fe, Ni); (3) the highly reducing local geochemical environment; and (4) the elevated temperatures sufficient for the maintenance of the reduction processes. The sources of phosphorus, iron and nickel can be inferred from the any geochemical environment similar to that described in this paper. The phosphate minerals related to the apatite group, Ca_5_(PO_4_)_3_(F,Cl,OH), are widespread both in meteorites and on the Earth; their terrestrial occurrence in the Hadean Eon has been discussed and looks undoubtful^31^. The same considerations can be applied to the source of Fe and Ni^31^: ferric-ferrous spinels (magnetite, magnesioferrite, chromite) and common sulfides like pyrite FeS_2_, pyrrhotite Fe_1-x_S, and pentlandite (Fe,Ni)_9_S_8_ could be likely precursors for Fe-Ni-phosphides. The fact that the metamorphic phosphide-bearing rocks described in this paper were primarily of sedimentary origin does not disrupt the overall geological reliability of the proposed model: the coexistence of apatite with Fe-spinels or sulfides is equally common in magamatic, metamorphic or sedimentary rocks (see for instance ref. 32).

The most debatable condition (3) implies the occurrence of strong reducing agent like the elemental carbon, methane or elemental hydrogen which are all known to be efficient phosphate reductants. It is known that the solid-state reduction of calcium phosphate by graphite begins above 1050°C^33^ whereas hydrogen-induced reduction of phosphates can be achieved at the temperatures above 400°C^34^. The possibility of reduction of phosphates by methane can not be ruled out^30^,^35^,^36^. Indeed, the speciation of carbon and hydrogen at the early Earth is widely debatable matter^35^,^36^,^37^,^38^,^39^ but, notwithstanding the diversity of existing opinions it is apparent that the geochemical environment in the Hadean and early Archaen Eons has been much more reduced than contemporary one^35^,^36^,^37^,^38^,^39^. Therefore it looks quite likely that elevated concentrations of elemental carbon, CH_4_ or H_2_ could be occurred at the local terrestrial sites at the Earth crust faults and volcanic areas. The elevated temperatures (condition 4) required for phosphate reduction could also be reached at the same environment^40^; however, other high-temperature natural events such as lightnings^18^,^19^,^41^ or combustion of abiogenic hydrocarbons^35^,^36^ can not be ruled out.

Phosphides are known as readily oxidizible minerals^1^,^2^,^3^,^4^,^5^. That is clearly supported by our findings showing that the diverse phosphide minerals from Levant were subject to pronounced surface oxidation. Therefore one can hardly expect that the unstable Earth's phosphides even emerged in the Hadean and Archaen Eons could be survived under late oxidizing environment on the geological timescale. Instead, they would be oxidatively consumed providing a source of phosphinites and phosphites: a likely basis for the geosynthesis of prebiotic organophosphorus compounds on the Earth^1^,^2^,^3^,^4^,^5^.

## Methods

The mineral samples used for the study in this work were collected during the field trips in 2005–2013 across the localities of the Hatrurim Formation (Michail Murashko and Yevgeny Vapnik), the coordinates of the localities were determined during the field works.

Electron microprobe analyses of the minerals were performed in energy dispersive mode (acceleration voltage 20 kV, beam current 0.5–1 nA) by means of JEOL JSM 6460 LV scanning electron microscope equipped with Oxford Instruments INCA X-sight LN2 EDS system; analytical standards: pyrite (Fe and S), metallic Co (Co), metallic Ni (Ni), GaP (P), metallic Cr (Cr). Representative chemical compositions of phosphides are given in Supplementary tables S1–S7.

X-ray single crystal studies were carried out using Bruker APEX DUO CCD diffractometer (for negevite, zuktamrurite, halamishite and transjordanite) and Stoe IPDS II image plate diffractometer (for murashkoite). Crystallographic data, details of the data collection and structure refinements are given in Supplementary tables S8–S12.

Supplementary information includes materials and methods, chemical compositions of studied phosphides (Tables S1–S7) and crystallographic data on phosphides (Tables S8–S12).

## Author Contributions

S.N.B. designed the work, performed single-crystal studies and wrote the manuscript. M.N.M. carried out field work and performed EDX measurements. Ye.V. provided geological background for the study and carried out field work. Yu.S.P. prepared the samples for study and performed EDX measurements. S.V.K. wrote the manuscript. All authors discussed the results.

## Supplementary Material

Supplementary Information Supplementary Material

## Figures and Tables

**Table 1:** Summary of known natural Fe-Ni-P phosphides arranged in order of increasing metal-to-phosphorus atomic ratio. Minerals found in the rocks of Hatrurim Formation are highlighted in italic, new minerals – in bold and italic. Estimated standard deviations of lattice parameters (Å) are given in parentheses. Data for allabogdanite from meteorite Onello^11^, nickelphosphide from meteorite Butler (present study), melliniite from meteorite Northwest Africa 1054 (ref 13), other phosphides from the Hatrurim Formation (present study). Reference numbers of the new minerals approved by the Commission on New Minerals, Nomenclature and Classification of International Mineralogical Association: negevite, 2013–104; zuktamrurite, 2013–107; murashkoite, 2012–071; halamishite, 2013–105; transjordanite, 2013–106

**Mineral**	**Formula**	**Structuretype**	**Crystalsystem**	**Spacegroup**	**Lattice parameters**			**Occurrence Earth Meteorites**	
					***a***	***b***	***c***		
***Negevite***	NiP_2_	Pyrite	Cub.	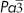	5.4816(5)			**+**	
***Zuktamrurite***	FeP_2_	Marcasite	Orth.	*Pnnm*	4.9276(6)	5.6460(7)	2.8174(4)	**+**	
***Murashkoite***	FeP	MnP	Orth.	*Pnma*	5.098(5)	3.251(1)	5.699 (3)	**+**	
***Halamishite***	Ni_5_P_4_	Ni_5_P_4_	Hex.	*P*6_3_*mc*	6.8184(4)		11.0288(8)	**+**	
***Transjordanite***	Ni_2_P	Fe_2_P	Hex.	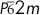	5.8837(3)		3.3492(4)	**+**	**?**
*Barringerite*	Fe_2_P	Fe_2_P	Hex.	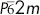	5.867(1)		3.465(1)	**+**	**+**
Allabogdanite	(Fe,Ni)_2_P	Co_2_Si	Orth.	*Pnma*	5.792(7)	3.564(4)	6.691(8)		**+**
Nickelphosphide	(Ni,Fe)_3_P	Fe_3_P	Tetr.	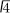	9.021(1)		4.4539(8)		**+**
*Schreibersite*	Fe_3_P	Fe_3_P	Tetr.	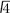	9.103(3)		4.461(2)	**+**	**+**
Melliniite	(Ni,Fe)_4_P	Au_4_Al	Cub.	*P*2_1_3	6.025(1)				**+**
