# Supplementary material for: Earth's Phosphides in Levant and insights into the source of Archean prebiotic phosphorus
Source: Sci Rep. 2015 Feb 10;5:8355. doi: 10.1038/srep08355 (PMC4322360; doi:10.1038/srep08355)
Supplement: Supplementary Information — Supplementary Material [file srep08355-s1.pdf]

Supplementary Materials

to the article:

Earth's Phosphides in Levant and insights into the source of Archean prebiotic phosphorus

Sergey N. Britvin, Michail N. Murashko, Yevgeny Vapnik, Yury S. Polekhovsky and Sergey V. Krivovichev

Table S1. Chemical composition of barringerite, $(Fe,Ni)_2P$, from the localities of the Hatrurim Formation.

**Element, wt. % ***				**Formula amounts based on 3 atoms per formula unit**			**Locality #**
**Fe**	**Ni**	**P**	**Total**	**Fe**	**Ni**	**P**	
55.5	21.2	22.3	99.0	1.44	0.52	1.04	1
54.6	22.2	22.3	99.1	1.41	0.55	1.04	1
65.3	10.5	22.6	98.4	1.69	0.26	1.05	2
78.7	0.0	22.2	100.9	1.99	0.00	1.01	2
76.6	3.2	21.0	100.8	1.96	0.08	0.97	2
74.5	4.7	22.1	101.3	1.88	0.11	1.01	2
74.9	4.1	22.1	101.1	1.89	0.10	1.01	2
79.1	0.0	22.1	101.2	1.99	0.00	1.00	1
75.1	3.6	22.2	100.9	1.90	0.09	1.01	1
72.5	3.4	22.7	98.6	1.86	0.08	1.05	1
76.9	2.3	22.1	101.3	1.94	0.06	1.01	1
76.0	2.5	22.2	100.7	1.93	0.06	1.01	1
75.9	0.0	23.6	99.5	1.92	0.00	1.08	3
69.8	6.7	23.1	99.6	1.78	0.16	1.06	3
16.7	60.6	22.4	99.7	0.44	1.51	1.06	3
71.3	5.2	22.7	99.2	1.83	0.13	1.05	3
76.3	1.0	21.4	98.7	1.98	0.02	1.00	1

Contents of Co are below detection limit (<0.1 wt.%)

Table S2. Chemical composition of transjordanite, $(Ni,Fe)_2P$, from the localities of the Hatrurim Formation.

**Element, wt. % ***				**Formula amounts based on 3 atoms per formula unit**			**Locality #**
**Fe**	**Ni**	**P**	**Total**	**Fe**	**Ni**	**P**	
22.7	54.8	21.9	99.4	0.60	1.37	1.04	1
20.5	56.8	21.9	99.2	0.54	1.42	1.04	1
10.1	68.8	21.5	100.4	0.27	1.72	1.02	1
34.1	42.9	22.1	99.1	0.89	1.07	1.04	1
10.3	67.7	21.7	99.7	0.27	1.70	1.03	1
5.4	73.0	21.6	100.0	0.14	1.83	1.03	1
1.2	77.1	21.6	99.9	0.03	1.94	1.03	1
10.6	67.0	21.7	99.3	0.28	1.69	1.03	1
11.8	66.4	21.7	99.9	0.31	1.66	1.03	1
9.4	68.3	21.7	99.4	0.25	1.72	1.03	1
15.8	61.9	21.8	99.5	0.42	1.55	1.03	3
31.9	48.6	20.7	101.2	0.83	1.20	0.97	1
4.3	75.6	21.3	101.2	0.11	1.88	1.01	1
11.3	68.3	21.4	101.0	0.30	1.70	1.01	1
6.7	73.0	21.1	100.8	0.18	1.82	1.00	1
16.0	60.9	22.9	99.8	0.42	1.51	1.07	3
17.1	60.5	21.6	99.2	0.45	1.52	1.03	3
8.5	69.6	22.4	100.5	0.22	1.73	1.05	3
8.5	69.6	21.3	99.4	0.23	1.76	1.02	3
16.7	60.6	22.4	99.7	0.44	1.51	1.06	3
10.2	67.8	21.5	99.5	0.27	1.71	1.02	3

Contents of Co are below detection limit (<0.1 wt.%)

Table S3. Chemical composition of murashkoite, FeP, from the localities of the Hatrurim Formation.

Element, wt. % *				Formula amounts based on 2 atoms per formula unit			Locality #
Fe	Ni	P	Total	Fe	Ni	P	
52.8	10.0	36.8	99.6	0.82	0.15	1.03	2
59.5	1.9	37.2	98.6	0.93	0.03	1.04	2
57.4	4.3	37.1	98.8	0.89	0.06	1.04	2
47.4	15.3	36.7	99.4	0.74	0.23	1.03	2
61.5	0.0	37.2	98.7	0.96	0.00	1.04	2
62.7	0.6	36.8	100.1	0.97	0.01	1.02	2
61.7	0.4	37.0	99.1	0.96	0.01	1.04	1
63.2	0.9	36.6	100.7	0.97	0.01	1.01	1
52.1	10.8	36.7	99.6	0.81	0.16	1.03	1
61.1	0.8	37.1	99.0	0.95	0.01	1.04	2
61.4	0.0	37.2	98.6	0.96	0.00	1.04	2
46.0	16.2	36.8	99.0	0.72	0.24	1.04	2
54.6	7.6	37.0	99.2	0.85	0.11	1.04	2
57.2	7.4	36.4	101.0	0.88	0.11	1.01	2
60.0	2.3	37.0	99.3	0.93	0.03	1.04	2
44.4	17.9	36.8	99.1	0.69	0.27	1.04	1
62.6	0.0	36.9	99.5	0.97	0.00	1.03	1
45.7	16.8	36.8	99.3	0.71	0.25	1.04	2
59.3	2.2	37.2	98.7	0.92	0.03	1.04	2
61.6	0.4	37.1	99.1	0.96	0.01	1.04	2
59.0	3.5	36.9	99.4	0.92	0.05	1.03	2
57.5	5.2	36.9	99.6	0.89	0.08	1.03	2
60.2	1.6	37.2	99.0	0.93	0.02	1.04	2
60.7	2.8	36.7	100.2	0.94	0.04	1.02	2
64.7	0.0	36.5	101.2	0.99	0.00	1.01	2
61.3	3.8	36.3	101.4	0.94	0.06	1.00	2
64.5	3.3	33.3	101.1	1.01	0.05	0.94	1
50.2	15.6	36.0	101.8	0.77	0.23	1.00	1
47.2	18.0	36.1	101.3	0.73	0.26	1.01	1
45.2	21.4	32.4	99.0	0.73	0.33	0.94	1
63.1	1.2	36.6	100.9	0.97	0.02	1.01	1
48.4	16.6	36.2	101.2	0.75	0.24	1.01	2
57.0	8.3	36.3	101.6	0.87	0.12	1.00	2

Contents of Co are below detection limit (<0.1 wt.%)

Table S3 (continued). Chemical composition of murashkoite, FeP, from the localities of the Hatrurim Formation.

Element, wt. % *				Formula amounts based on 2 atoms per formula unit			Locality #
Fe	Ni	P	Total	Fe	Ni	P	
63.7	1.2	36.4	101.3	0.98	0.02	1.01	2
64.4	0.6	35.4	100.4	1.00	0.01	0.99	2
64.7	0.3	36.4	101.4	0.99	0.00	1.00	2
62.7	2.3	36.0	101.0	0.97	0.03	1.00	2
61.0	4.1	35.0	100.1	0.95	0.06	0.99	2
64.9	0.0	34.8	99.7	1.02	0.00	0.98	2
63.5	0.0	35.5	99.0	1.00	0.00	1.00	3
60.8	0.0	37.7	98.5	0.94	0.00	1.06	3
44.7	17.4	37.1	99.2	0.70	0.26	1.04	3
60.0	4.0	38.3	102.3	0.90	0.06	1.04	3
58.4	6.1	37.0	101.5	0.89	0.09	1.02	3
47.4	14.4	38.2	100.0	0.73	0.21	1.06	3
55.0	7.3	38.4	100.7	0.84	0.11	1.06	3
56.7	5.9	36.0	98.6	0.89	0.09	1.02	3
61.9	0.0	36.7	98.6	0.97	0.00	1.03	3
59.8	2.4	38.6	100.8	0.91	0.03	1.06	3
57.2	3.4	36.4	97.0	0.91	0.05	1.04	3
61.7	0.0	37.9	99.6	0.95	0.00	1.05	3
63.5	0.0	35.5	99.0	1.00	0.00	1.00	3
60.8	0.0	38.8	99.6	0.93	0.00	1.07	3
44.7	17.4	36.7	98.8	0.70	0.26	1.04	3
56.8	4.8	37.7	99.3	0.88	0.07	1.05	3
52.8	8.2	38.6	99.6	0.81	0.12	1.07	3
64.7	0.0	36.4	101.1	0.99	0.00	1.01	3
64.5	0.4	34.4	99.3	1.02	0.01	0.98	3
61.3	0.4	37.5	99.2	0.95	0.01	1.05	3
58.5	3.4	37.0	98.9	0.91	0.05	1.04	3
58.0	4.8	36.5	99.3	0.90	0.07	1.03	3
61.3	1.0	37.0	99.3	0.95	0.01	1.03	3
63.8	0.9	35.6	100.3	0.99	0.01	1.00	1

Contents of Co are below detection limit (<0.1 wt.%)

Table S4. Chemical composition of negevite, NiP_2, from wadi Nahal Halamish (locality #1), Hatrurim Formation

Element, wt. %						Formula amounts based on 3 atoms per formula unit				
**Fe**	**Ni**	**Co**	**P**	**S**	**Total**	**Fe**	**Ni**	**Co**	**P**	**S**
3.2	42.5	3.7	43.2	8.0	100.6	0.07	0.87	0.08	1.68	0.30
2.5	42.0	3.1	43.1	8.8	99.5	0.05	0.87	0.06	1.68	0.33
2.9	43.2	3.4	42.5	8.2	100.2	0.06	0.89	0.07	1.66	0.31

Table S5. Chemical composition of zuktamrurite, FeP_2, from wadi Nahal Halamish (locality #1), Hatrurim Formation

Element, wt. % *				Formula amounts based on 3 atoms per formula unit		
**Fe**	**Ni**	**P**	**Total**	**Fe**	**Ni**	**P**
39.9	7.8	51.4	99.1	0.86	0.16	1.99
40.5	8.4	51.8	100.7	0.86	0.17	1.97
39.7	7.7	51.9	99.3	0.85	0.16	2.00

Contents of Co are below detection limit (<0.1 wt.%)

Table S6. Chemical composition of halamishite, Ni_5P_4, from wadi Nahal Halamish (locality #1), Hatrurim Formation

Element, wt. % *				Formula amounts based on 9 atoms per formula unit		
**Fe**	**Ni**	**P**	**Total**	**Fe**	**Ni**	**P**
1.6	68.9	29.7	100.2	0.12	4.89	5.01
1.9	69.4	29.5	100.8	0.14	4.91	5.05
1.9	69.2	29.7	100.8	0.14	4.89	5.03

Contents of Co are below detection limit (<0.1 wt.%)

Table S7. Chemical composition of schreibersite, $(Fe,Ni)_3P$, from the localities of the Hatrurim Formation.

Element, wt. %						Formula amounts based on 4 atoms per formula unit					Locality #
Fe	Ni	Co	Cr	P	Total	Fe	Ni	Co	Cr	P	
81.3	1.5	0.2	0.4	15.0	98.4	2.95	0.05	0.01	0.02	0.98	2
81.8	1.6	0.4	0.4	14.6	98.8	2.96	0.06	0.01	0.02	0.95	2
82.9	1.4	b.d.l.	0.5	15.0	99.8	2.97	0.05		0.02	0.97	1
83.7	b.d.l.	b.d.l.	b.d.l.	15.8	99.5	2.98				1.02	3

Table S8. Crystal parameters, data collection and structure refinement details for the crystal of negevite.

Crystal Data	
Chemical formula	$(Ni_{0.89}Co_{0.07}Fe_{0.06})_{1.00}(P_{1.69}S_{0.31})_{2.00}$
Crystal size (mm)	0.01 x 0.01 x 0.01
Crystal system	Cubic
Space group	$Pa\overline{3}$
a (Å)	5.4816(5)
V (Å^3)	164.71(3)
Z	4
D_x (g/cm^3)	4.881(1)
Data Collection	
Instrument	Bruker APEX DUO (CCD detector)
X-ray source and optics	Mo$K\alpha$ (λ = 0.71073 Å), microfocus tube, 50 kV, 0.6 mA
Average temperature (K)	293
Detector to sample distance (mm)	37.6
Frame width (degrees)	1
Number of frames	672
Exposure per frame (s)	80
2Θ range (degrees)	12.90 – 60.86
Total collected reflections	2140
Unique reflections	84
Unique observed $\lvert F_o \rvert \geq 4\sigma_F$	67
$R_{int.}$	0.073
R_σ	0.031
hkl range	$-7 \leq h \leq 7$; $-7 \leq k \leq 7$; $-7 \leq l \leq 7$
Refinement	
Reflection file type	HKLF 4
R_1 ($\lvert F_o \rvert \geq 4\sigma_F$)	0.024
R_1 (all data)	0.035
wR_2	0.042
$S=GooF$	1.053

Table S9. Crystal parameters, data collection and structure refinement details for the crystal of zuktamrurite.

Crystal Data	
Chemical formula	$(Fe_{0.84}Ni_{0.16})_{1.00}P_{2.00}$
Crystal size (mm)	0.01 x 0.01 x 0.01
Crystal system	Orthorhombic
Space group	*Pnnm*
a (Å)	4.9276(6)
b (Å)	5.6460(7)
c (Å)	2.8174(4)
V (Å^3)	78.38(1)
Z	2
D_x (g/cm^3)	5.014(1)
Data Collection	
Instrument	Bruker APEX DUO (CCD detector)
X-ray source and optics	Mo$K\alpha$ (λ = 0.71073 Å), microfocus tube, 50 kV, 0.6 mA
Average temperature (K)	293
Detector to sample distance [illegible]	37.6
Frame width (degrees)	1
Number of frames	864
Exposure per frame (s)	180
2Θ range (degrees)	10.98 – 50.72
Total collected reflections	481
Unique reflections	80
Unique observed $\|F_o\| \geq 4\sigma_F$	65
$R_{int.}$	0.070
R_σ	0.047
hkl range	$-5 \leq h \leq 4$; $-5 \leq k \leq 6$; $-3 \leq l \leq 3$
Refinement	
Reflection file type	HKLF 4
R_1 ($\|F_o\| \geq 4\sigma_F$)	0.051
R_1 (all data)	0.059
wR_2	0.129
$S = GooF$	1.191

Table S10. Crystal parameters, data collection and structure refinement details for the crystal of murashkoite.

**Crystal Data**	
Formula	FeP
Crystal size (mm)	0.05 x 0.06 x 0.12
Crystal system	Orthorhombic
Space group	*Pnma*
a (Å)	5.099(2)
b (Å)	3.251(2)
c (Å)	5.695(2)
V ($Å^3$)	94.41(8)
Z	4
D_x (g/cm^3)	6.108(5)
**Data Collection**	
Instrument	Stoe IPDS II (image plate detector)
Radiation	Mo*K*α (λ = 0.71073 Å)
Average temperature (K)	273
Detector to sample distance (mm)	80
Omega increment (degrees)	2
Number of frames	90
Exposure per frame (min)	8
2Θ range (degrees)	7.2 - 56.00
Total collected reflections	1504
Unique reflections	129
Unique observed $\|F_o\| \geq 4\sigma_F$	119
$R_{int.}$	0.170
R_σ	0.017
hkl range	$-6 \leq h \leq 6$; $-4 \leq k \leq 4$; $-7 \leq l \leq 7$
**Refinement**	
Reflection file type	HKLF 4
R_1 ($\|F_o\| \geq 4\sigma_F$)	0.050
R_1 (all data)	0.054
wR_2	0.149
S=GooF	1.419

Table S11. Crystal parameters, data collection and structure refinement details for the crystal of halamishite.

Crystal Data	
Simplified formula	Ni_5P_4
Crystal size (mm)	0.01 x 0.01 x 0.01
Crystal system	Hexagonal
Space group	$P6_3mc$
a (Å)	6.8184(4)
c (Å)	11.0288(8)
V (Å^3)	444.04(5)
Z	4
D_x (g/cm^3)	6.244(1)
Data Collection	
Instrument	Bruker APEX DUO (CCD detector)
X-ray source and optics	Mo$K\alpha$ (λ = 0.71073 Å), microfocus tube, 50 kV, 0.6 mA
Average temperature (K)	293
Detector to sample distance (mm)	37.6
Frame width (degrees)	1
Number of frames	607
Exposure per frame (s)	60
2Θ range (degrees)	12.90 – 60.86
Total collected reflections	5494
Unique reflections	425
Unique observed $\|F_o\| \geq 4\sigma_F$	391
$R_{int.}$	0.074
R_σ	0.039
hkl range	$-8 \leq h \leq 8$; $-8 \leq k \leq 8$; $-14 \leq l \leq 14$
Refinement	
Reflection file type	HKLF 4
R_1 ($\|F_o\| \geq 4\sigma_F$)	0.027
R_1 (all data)	0.037
wR_2	0.062
$S = GooF$	1.287

Table S12. Crystal parameters, data collection and structure refinement details for the crystal of transjordanite

Crystal Data	
Chemical formula	$(Ni_{1.72}Fe_{0.28})_2P_{1.00}$
Crystal size (mm)	0.05 x 0.06 x 0.08
Crystal System	Hexagonal
Space Group	$P\bar{6}2m$
a (Å)	5.8897(3)
c (Å)	3.3547(2)
V (Å^3)	100.78(1)
Z	3
D_x (g/cm^3)	7.297(1)
Data Collection	
Instrument	Bruker APEX DUO (CCD detector)
X-ray source and optics	Mo$K\alpha$ (λ = 0.71073 Å), microfocus tube, 50 kV, 0.6 mA
Average temperature (K)	293
Detector to sample distance (mm)	37.6
Frame width (degrees)	0.5
Number of frames	3438
Exposure per frame (s)	3
2Θ range (degrees)	4.00 – 52.59
Total collected reflections	6721
Unique reflections	469
Unique observed $\|F_o\|\geq4\sigma_F$	443
$R_{int.}$	0.071
R_σ	0.030
hkl range	$-13 \leq h \leq 12$; $-12 \leq k \leq 12$; $-7 \leq l \leq 6$
Refinement	
Reflection file type	HKLF 4
R_1 ($\|F_o\|\geq4\sigma_F$)	0.019
R_1 (all data)	0.022
wR_2	0.037
$S=GooF$	1.115
